# Turning the tide on organ fibrosis: a special issue on scientific advances and next-generation treatments

**DOI:** 10.1172/jci.insight.210035

**Published:** 2026-07-08

**Authors:** Melanie Königshoff, Gavin Arteel, Corinne Williams, Oliver Eickelberg

We are delighted to present our first thematic issue of *JCI Insight* today, which focuses on tissue fibrosis across organ systems, broadly defined as the excessive accumulation of ECM components as a pathological response to chronic tissue injury or inflammation. The word fibrosis is derived from the Latin word fibra (fiber) and the Greek suffix -osis (condition/process). Ancient descriptions of tissue fibrosis include Egyptian medicine (1600 BCE or earlier, mostly describing scar formation in surgical contexts) and Greek records from the Hippocratic Corpus (around 400 BCE, describing tissue hardening and excess connective tissue accumulation). It is widely accepted that the first modern medical description of tissue fibrosis is attributed to English surgeon and pathologist John Browne, who provided the earliest known clinical assessment of a fibrotic organ by describing liver cirrhosis in an autopsy as a “glandulous-appearing liver” in 1685 (2). Today, we know that fibrosis can occur in almost every organ of the human body. It is often diagnosed in the skin, lung, heart, vasculature, kidney, and liver, among other organs. Organ fibrosis almost always critically compromises normal organ function. Fibrosis, once established, is also a major driver of morbidity and mortality worldwide, representing one of the most formidable and unresolved challenges in modern medicine. Given the broad range of organs affected by fibrotic disease, it is essential to understand both common and organ-specific mechanisms that underlie fibrosis onset, progression, and resolution to aid the development of novel therapeutic approaches. 

It is thought that fibrosis arises as a dysregulated wound-healing response to chronic injury or inflammation, which leads to excessive deposition of ECM components that interfere with normal organ architecture and progressively impair functionality. Despite decades of research, fundamental pathomechanisms — such as the precise triggers for the shift from resolution to relentless progression, the heterogeneity of fibroblast activation, and the limited regenerative capacity of fibrotic tissue — remain incompletely understood, resulting in a striking paucity of effective disease-modifying therapies.

Current treatments are largely supportive or modestly antifibrotic at best. Despite more recent successes, many clinical trials have failed owing to the multifactorial nature fibrotic disease, persistent difficulties in early diagnosis due to a paucity of suitable biomarkers, or the notorious irreversibility of advanced scarring. This therapeutic gap carries profound clinical and societal consequences. Fibroproliferative disorders are estimated to contribute to nearly 45% of deaths in developed nations, driving substantial morbidity, reduced quality of life, and enormous economic burdens through hospitalizations, chronic care, lost productivity, and high-cost interventions, such as organ transplantation (which to date represent the only potential cure for several fibrotic disorders). For instance, in idiopathic pulmonary fibrosis alone, annual per-patient costs can exceed tens of thousands of dollars (often $20,000–$100,000+, depending on the healthcare system and disease stage), with an estimated contribution of around $2 billion annually to the US healthcare system in medical expenses. Addressing these challenges demands discovery of deeper mechanistic insights, identification of innovative biomarkers, and/or multimodal or even combination therapy strategies to truly freeze or reverse tissue fibrosis before irreversible organ failure sets in.

At a fundamental and mechanistic level, organ fibrosis involves the persistent, abnormal activation of quiescent fibroblasts that interact with resident parenchymal and/or immune cells through complex signaling cascades. Recent advances in multi-omics technologies have revealed that fibrosis is a dynamic process spanning the molecular, cellular, microenvironmental, and organ levels, with key cellular players, including epithelial cells, fibroblasts, and immune cells, all contributing to disease progression. Emerging research has also highlighted cellular senescence, mechanotransduction, and the underestimated heterogeneity and plasticity of previously thought uniform cell populations as important drivers of fibrogenesis. Alongside organ-specific mechanisms of fibrosis, investigators have increasingly recognized shared core pathways across several organs, fostering interest in developing pan-organ antifibrotic drugs.

This issue features a state-of-the-art compendium of fibrosis research, selected via rigorous peer review and editorial priorities We believe that we have selected the best articles from this call, covering a range of organ systems, including lung, skin, heart, kidney, liver, bone, and the gastrointestinal tract, and diseases, including cancer and HIV. These contributions come from labs across the US, Europe, and Asia, further highlighting the global burden of fibrosis. In addition, these articles are framed by five outstanding Reviews from leaders in the fibrosis field, which address current knowledge and recent advances in this rapidly growing field.

Yang, Steffani, and colleagues focus on the newly discovered heterogeneity of fibroblasts and their role in homeostasis, regulating tissue maintenance, physiological wound healing, and regeneration. They discuss how aberrant fibroblast activation underlies the hallmarks of maladaptive repair across organs and in cancer.

Humphreys walks us through new concepts and shared mechanisms of organ fibrosis that have emerged from recent multi-omic approaches, focusing on novel signaling pathways linked to the underlying dysfunction that drive ECM accumulation. These shared pathways may hold the key to therapeutic approaches and, with artificial intelligence playing an increasingly important role in target and drug discovery, may pave the way for an increasingly personalized therapeutic approach. 

On the therapeutic front, the pipeline of potential antifibrotics has expanded considerably: current strategies encompass small-molecule inhibitors, monoclonal antibodies targeting fibrosis mediators, gene therapies, and cell-based approaches. In addition, innovative drug delivery systems and pulsed magnetic field therapies are opening new avenues for more precise treatment. Along these lines, Lebeaupin and colleagues provide important insight into the future of antifibrotic therapy development and the role of the ‘omics revolution in identifying and validating promising targets. Desert et al. highlight ECM components of the tumor stroma as potential therapeutic targets for reversing fibrosis.

Historically, fibrosis has been considered permanent, with therapeutics aimed at arresting disease and preventing progression. However, we are now entering an exciting phase, in which reversal of fibrosis and regeneration of tissue function, while still ambitious, are potentially feasible aims. Greven et al. discuss the inflammatory/fibrotic axis and advances in fibrosis reversal in myelofibrosis. This disease provides a powerful model for alleviating fibrosis in other organs.

Despite tremendous progress, a substantial translational gap remains between identifying antifibrotic targets and translating that knowledge into effective therapies for patients. In addition to these Reviews, this issue features exciting new research on fibrosis spanning a range of organ systems, including the lung, skin, heart, kidney, liver, bone, and the gastrointestinal tract, as well as diseases such as cancer and HIV.

Thank you to all the authors and reviewers, who contributed their time and expertise to this special issue. We are extremely grateful for your contributions. Fibrosis remains a challenging and critically important area of research, underscoring the need for continued interdisciplinary collaboration across research, clinical, and industry settings. The progress the field has made in understanding the underlying mechanisms of disease offers renewed hope for more therapeutic options.

## Version 1 07/08/2026

Electronic publication
